# Two distinct regions in the model protein Peb1 are critical for its heterologous transport out of *Escherichia coli*

**DOI:** 10.1186/1475-2859-9-97

**Published:** 2010-12-02

**Authors:** Lena Anton, Katariina Majander, Harri Savilahti, Liisa Laakkonen, Benita Westerlund-Wikström

**Affiliations:** 1 Division of General Microbiology, Department of Biosciences, P.O. Box 56, FIN-00014 University of Helsinki, Helsinki, Finland; 2 Division of Genetics and Physiology, Department of Biology, FIN-20014 University of Turku, Turku, Finland; 3 Division of Biochemistry, Department of Biosciences, P.O. Box 56, FIN-00014 University of Helsinki, Helsinki

## Abstract

**Background:**

*Escherichia coli *is frequently the first-choice host organism in expression of heterologous recombinant proteins in basic research as well as in production of commercial, therapeutic polypeptides. Especially the secretion of proteins into the culture medium of *E. coli *is advantageous compared to intracellular production due to the ease in recovery of the recombinant protein. Since *E. coli *naturally is a poor secretor of proteins, a few strategies for optimization of extracellular secretion have been described. We have previously reported efficient secretion of the diagnostically interesting model protein Peb1 of *Campylobacter jejuni *into the growth medium of *Escherichia coli *strain MKS12 (Δ*fliCfliD*). To generate a more detailed understanding of the molecular mechanisms behind this interesting heterologous secretion system with biotechnological implications, we here analyzed further the transport of Peb1 in the *E. coli *host.

**Results:**

When mature Peb1 was expressed without its SecA-YEG -dependent signal sequence and without the putative signal peptidase II recognition sequence in *E. coli *MKS111ΔHBB lacking the flagellar secretion complex, the protein was found in the periplasm and growth medium which indicated a flagellum-independent translocation. We assessed the Peb1 secretion proficiency by an exhaustive search for transport-affecting regions using a transposition-based scanning mutagenesis strategy. Strikingly, insertion mutagenesis of only two segments, called TAR1 (residues 42 and 43) and TAR2 (residues 173 to 180), prevented Peb1 secretion individually. We confirmed the importance of TAR regions by subsequent site-specific mutagenesis and verified that the secretion deficiency of Peb1 mutants was not due to insolubility or aggregation of the proteins in the cytoplasm. We found by cell fractionation that the mutant proteins were present in the periplasm as well as in the cytoplasm of MKS12. Hence, mutagenesis of TAR regions did not affect export of Peb1 across the cytoplasmic membrane, whereas its export over the outer membrane was markedly impaired.

**Conclusions:**

We propose that the localization of the model protein Peb1 in the growth medium of *E. coli *is due to active secretion by a still unknown pathway of *E. coli*. The secretion apparently is a two-step process involving a periplasmic step and the TAR regions.

## Background

Protein secretion is one of the main means by which bacteria interact with their environment. The interaction may take place in a variety of manners: bacteria secrete enzymes, toxins and other virulence factors, excrete metabolic waste products, and export binding proteins into the periplasm for import of nutrients or export of toxic compounds. Bacteria also use different secretion systems to assemble on their surface organelles for motility, adhesion and injection of effector molecules into host cells [1,2]. Bacterial protein secretion systems are of great importance from a virulence-associated viewpoint as potential targets for novel antibacterial drugs [3] but are significant also commercially and therapeutically due to the use of bacteria as protein factories for the delivery of polypeptides into bacterial growth medium [4,5].

In bacteria, secretion is regarded as active transport of proteins from the cytoplasm to the exterior of the cell [6]. In Gram-negative bacteria, the proteins to be exported have to first cross the cytoplasmic membrane (CM), usually at the cost of ATP hydrolysis, and further the outer membrane (OM). Six distinct protein secretion pathways have currently been described in Gram-negative bacteria, some of these to atomic detail [7-9]. Some of the secretion pathways, i. e. the type 2 and type 5 secretion systems consist of separate protein complexes on the two membranes [10-12], whereas others, i. e. the type 1, type 3, type 4 and type 6 pathways form continuous pores or tunnels crossing all the way from the cytoplasm to the exterior [13,14]. In addition to the complexes specifically devoted to protein secretion, bacteria possess so-called ATP-binding cassette (ABC) secretion systems, which carry a wide variety of substrates across the CM. Of these, the ABC importers catalyze the uptake of nutrients that are delivered to them by specific periplasmic substrate-binding proteins (PBPs), whereas ABC exporters are involved in the transport of e.g. drugs, lipopolysaccharides, toxins as well as proteins from the cytoplasm [13,15].

The food-borne human gastrointestinal pathogen *Campylobacter jejuni *expresses the protein Peb1 [16], which acts in distinct roles in separate compartments of the bacterial cell. First, Peb1 is present on the bacterial surface of all *C. jejuni *isolates analyzed, which makes it a putative target for diagnostics, and is regarded as a significant colonization and virulence factor. Antibodies against Peb1 are found in sera derived from the majority of patients convalescent from *C. jejuni *infection [16]. The protein mediates *C. jejuni *adherence *in vitro *to HeLa cells and is required for *C. jejuni *colonization of mice intestine [17,18]. Second, the major fraction of Peb1 is present in the periplasm of *C. jejuni *and acts as an aspartate/glutamate -binding PBP essential for optimal microaerobic growth of *C. jejuni *on these amino acids [19,20]. Finally, Peb1 is also found in minor amounts in the culture supernatant, but it is not present in the CM and OM [19]. For fulfilling each of its roles, Peb1 must be efficiently exported to the correct cell compartment. In the cytoplasm of *C. jejuni*, Peb1 carries an N-terminal signal sequence, typical of proteins secreted by the SecA-YEG pathway, which directs its export across the CM. A processing site typical for signal peptidase II has additionally been predicted in the N-terminus of Peb1, but the biological relevance of this processing site as well as the mechanism for the further transport of Peb1 from the periplasm across the OM in *C. jejuni *are still unknown [20].

We have previously shown that Peb1 expressed without its native SecA-YEG -dependent N-terminal secretion sequence and without the putative processing site for signal peptidase II is exported very efficiently into the growth medium at a high concentration and purity in an *Escherichia coli *flagellar mutant [21]. The reader is referred to Chevance and Hughes for a more detailed account on flagellar regulation and assembly [22]. Here we further analyze and document this phenomenon in order to increase the knowledge on how a heterologous model protein may be transported across the cell wall into the growth medium of *E. coli *and to broaden the understanding of factors that influence heterologous protein secretion in *E. coli*. We report that Peb1 is actively secreted in a flagellar-independent manner by an unknown two-step process and that the secretion is affected by mutagenesis of two distinct transport-affecting regions in Peb1.

## Results

### Peb1 is transported from *E. coli *MKS12 also independently of the flagellar secretion system

In a previous report, we showed that the *E. coli *strain MKS12 (Δ*fliC*Δ*fliD*), lacking the flagellar filament, is able to secrete recombinant flagellin (FliC), FliC fragments, FliC fusion proteins as well as heterologous proteins, like the model Peb1 protein, into the growth medium [21]. We also demonstrated that the extracellular location of these recombinant polypeptides was not a result of leakage from the periplasm or cytoplasm or lysis of the cells by showing the lack of the periplasmic maltose binding protein as well as the cytoplasmic proteins chloramphenicol acetyl transferase and GroEL in the growth medium.

**Figure 1:**
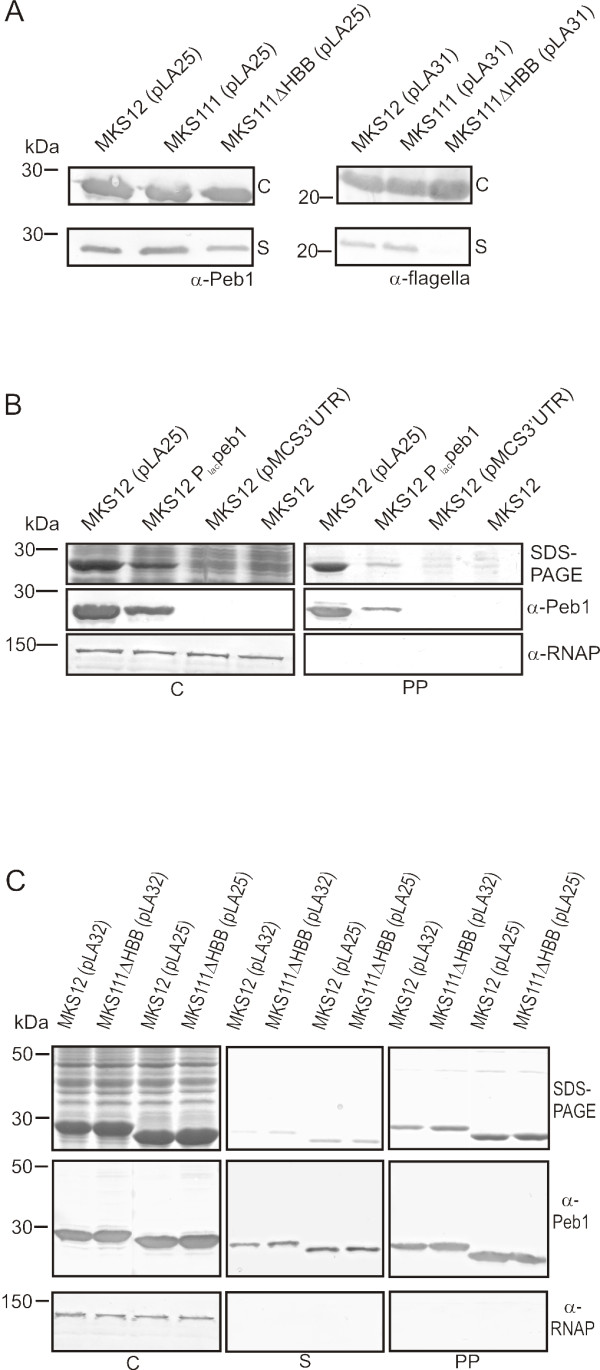
**Analysis of localization and secretion of expressed proteins in flagellar mutant strains**. (A) Western blot analyses with anti-Peb1 and anti-flagella antibodies of whole cells and cell-free culture supernatants from the strains indicated above the panels and expressing wt Peb1 (from pLA25) and FliC1-183 peptide (from pLA31), respectively. Note secretion of Peb1 in MKS111ΔHBB but lack of FliC1-183 secretion in the same strain. (B) Subcellular localization of Peb1 expressed from a plasmid or from the chromosome. Top panel shows SDS-PAGE and middle panel Western blot analysis with anti-Peb1 antibodies of whole -cell samples and periplasmic extracts of MKS12 (pLA25), MKS12P*_lac_peb1*, MKS12 (pMCS3'UTR), and MKS12. Lack of cell lysis and cytoplasmic leakage was controlled by immunoblotting with anti-RNAP antibodies (bottom panel). (C) SDS-PAGE (top panel) and Western blot (middle panel) analyses of whole cell, cell-free culture supernatant and periplasmic samples of strains MKS12 and MKS111ΔHBB carrying the plasmids pLA25 (encodes Peb1) and pLA32 (encodes FliC_1-20_Peb1). Lack of cytoplasmic leakage was controlled by immunoblotting with anti-RNAP antibodies (bottom panel). In all the panels of the figure, the letter C indicates whole-cell samples, S cell-free culture supernatants, and PP periplasmic extracts. All the samples correspond to 100 μl of culture and molecular weight markers in kDa are indicated on the left.

To generate a more detailed picture of the Peb1 transport event in *E. coli*, we here assessed the role of the flagellar export gate on Peb1 secretion. To construct a host strain lacking the flagellar export gate, the following modifications in strain MKS12 were made. We constructed the *E. coli *strains MKS111 and MKS111ΔHBB (see Table 1 for strains used), which are isogenic with strain MKS12; MKS111 carries additionally a deletion in the chromosomal gene encoding the transcriptional regulator FlgM. The *flgM *deletion releases the flagellar-specific sigma factor FliA, which is required for transcription from the P*_fliC _*promotor used in this study [23]. In strain MKS111ΔHBB, genes encoding flagellar basal body structures, i.e. the rings, the rod, the hook as well as components of the secretion apparatus, have additionally been deleted from the chromosome. Consequently, FliA-mediated transcription can proceed but flagellar filament components are not secreted from the cytoplasm across the membranes in strain MKS111ΔHBB. The Δ*fliC *gene of the strains MKS12, MKS111 and MKS111ΔHBB was complemented *in trans *with plasmids pLA25 and pLA31 (see Table 1 for plasmids used), which encode Peb1 without the sequences encoding its native N-terminal leader peptide and putative processing site for signal peptidase II, and the FliC1-183 peptide, respectively. Western blot analyses with anti-Peb1 and anti-flagella antibodies showed that Peb1 was present in cell-free culture supernatant of all the strains tested, whereas the FliC1-183 peptide, expressed similarly and used as a flagellum-secreted control [21], remained intracellular in MKS111ΔHBB (Figure 1A). The results showed that Peb1 is secreted to the extracellular milieu also in the strain lacking the flagellar export gate, whereas the C-terminally truncated flagellin and the full-length FliC (not shown) remain intracellular in the same strain. This finding indicated that the secretion of Peb1 in *E. coli *also can proceed independently of the flagellum.

**Table 1:** Bacterial strains and plasmids used in this study

Strain or plasmid	Relevant characteristics	Source or reference
Strains		
*E. coli *AAEC072A	MG1655 Δ*fimA-H*	[44]
*E. coli *MKS12	MG1655 Δ*fimA-H *Δ*fliCD*	[21]
*E. coli *MKS12P*_lac_peb1*	MKS12 carrying *peb1 *under P*_lac _*at the Δ*fliC *site	This study
*E. coli *MKS111	MG1655 Δ*fimA-H *Δ*fliCD *Δ*flgM*	This study
*E. coli *MKS111ΔHBB	MG1655 Δ*fimA-H *Δ*fliCD *Δ*flgM *Δ*fliFGH *Δ*flgDEFGHIJ*	This study
*E. coli *DH10B	*endA*- *RecA*- *Dam/Dcm*+	[66]
Plasmids		
pMCS3'UTR	Multiple cloning site preceding the 3'UTR sequence of *fliC *in *Pvu*I-*EcoR*I of pBR322	[21]
pPeb15'3'	173 bp *fliC *promoter sequence and 702bp fragment encoding mature Peb1 in *Pvu*I-*Sca*I site of pMCS3'UTR	[21]
pLA25	P*fliC *and fragment encoding mature Peb1, named *fliC*5'UTR-*peb1*, in *BglI*I-*Xba*I site of pMCS3'UTR	This study
pLA26	3'UTR removed by digestion at *Sca*I-*EcoR*I sites of pLA25	This study
pLA31	Fragment containing P*fliC *and truncated *fliC *(encoding N-terminal 183 aa) in *BglI*I-*Xba*I site of pMCS3'UTR	This study
pLA32	Fragment containing P*fliC*, truncated *fliC *(encoding N-terminal 20 aa) and peb1 (without ss) in *BglI*I-*Xba*I site of pLA25	This study
pTAR15G	Insertion of five glycines at residue D42 of Peb1. pLA25 background.	This study
pTAR1P	Substitution of four separate polar residues in Peb1 TAR1 region with alanine. pLA25 background.	This study
pTAR2P	Substitution of four separate polar residues in Peb1 TAR2 region with alanine pLA25 background.	This study
pTAR1A	Substitution of seven residues in Peb1 TAR1 region with alanine. pLA25 background.	This study
pTAR1G	Substitution of seven residues in Peb1 TAR1 region with glycine. pLA25 background.	This study
pTAR2A	Substitution of eight residues in Peb1 TAR2 region with alanine. pLA25 background.	This study
pTAR2G	Substitution of eight residues in Peb1 TAR2 region with glycine. pLA25 background.	This study

### Peb1 is present in the periplasmic space of *E. coli*

The finding that Peb1 is present in the growth medium also in a mutant lacking the flagellar transport complex prompted us to further analyze the phenomenon. To compare the localization of Peb1 expressed from a multicopy plasmid and from a single-copy gene from the chromosome, we first constructed the strain MKS12P*_lac_peb1*, which carries *peb1 *without signal sequences under a constitutive *lac *promoter (P*_lac_*) that lacks the *lac *operator region of P*_lac _*in the *fliC *deletion site on the chromosome of MKS12. We then prepared periplasmic fractions and cell samples of the *E. coli *strains MKS12 (pLA25) and MKS12P*_lac_peb1*, both expressing Peb1 without its native N-terminal signal sequences. SDS-PAGE and Western blot analysis with anti-Peb1 antibodies showed that the cellular concentration of Peb1 was approximately 50% lower in MKS12P*_lac_peb1 *than in MKS12 (pLA25), but the protein was clearly present in the periplasm in both strains. We estimated on the basis of SDS-PAGE analysis that the percentage of intracellular Peb1 found in the periplasm was 50% in MKS12 (pLA25) and 25% in MKS12P*_lac_peb1*. Peb1 was not detected in the control strains MKS12 and MKS12 (pMCS3'UTR) (Figure 1B). Western blot analysis with antibodies against the intracellular RNA polymerase β subunit (RNAP) indicated that the periplasmic fractionation was successful and that the presence of Peb1 in the periplasmic samples was not due to cytoplasmic contamination (bottom panel in Figure 1B). The results thus indicated that mature Peb1 is exported to the periplasm in *E. coli *and that the localization of Peb1 in the periplasm is apparently not related to leakage of significantly overexpressed protein.

To study the impact of the SecA-YEG pathway in Peb1 export, we analyzed periplasmic and cell samples of MKS12 (pLA25) grown in the presence and absence of subinhibitory concentration of azide, which is known to arrest SecA-YEG dependent export across the CM [24]. Results from SDS-PAGE analysis and Western blotting with anti-Peb1 antibodies revealed that the presence of Peb1 in the cytoplasm and the periplasm was not affected by growth in the presence of 3 mM NaN_3 _(data not shown). The data thus indicate that the SecA-YEG pathway may not be involved in the export of Peb1 across the CM.

### Fusion of a FliC fragment to the N-terminus of Peb1 does not affect secretion

We next assessed whether the N-terminus of Peb1 is involved in the secretion process, as has been reported for flagellar proteins and effector proteins in type 3 secretion systems [22,25]. The plasmid pLA32 encoding the fusion protein FliC_1-20_Peb1 was constructed and the protein expressed in the *E. coli *strains MKS12 and MKS111ΔHBB. SDS-PAGE and Western blot analysis with anti-Peb1 antibodies showed that the intracellular expression and extracellular secretion of FliC_1-20_Peb1 was at the same level as those of Peb1. FliC_1-20_Peb1 and Peb1 were also present in almost equal quantities in the periplasm of all the strains studied (Figure 1C). The periplasmic location of Peb1 and FliC_1-20_Peb1 was not due to cytoplasmic contamination as indicated by the Western blot analysis with antibodies against the intracellular protein RNAP (Figure 1C). The results thus revealed that fusion of a heterologous protein fragment to the processed N-terminus of Peb1 does not interfere with Peb1 secretion in MKS12 and MKS111ΔHBB, and that this fusion protein is secreted equally efficiently in both strains.

### Exhaustive search for transport-affecting regions in Peb1

**Figure 2:**
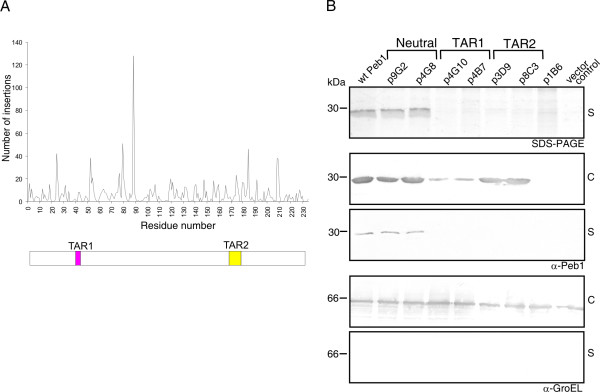
**Mutagenesis of Peb1 as well as analysis of Peb1 expression and secretion in representative mutant library clones**. (A) Distribution of the five-residue MGS-generated insertions in Peb1 and schematic presentation of Peb1 with the transport-affecting regions TAR1 (pink) and TAR2 (yellow) identified on the basis of the transposon mutagenesis indicated. (B) SDS-PAGE analysis of cell-free culture supernatant of MKS12 carrying the plasmids indicated (top panel). Western blot analysis with anti-Peb1 antibodies shows the level of Peb1 expression in whole bacterial cells of the various clones (second panel from the top) and verifies that the secreted protein is Peb1 (third panel from the top). Western blot analysis with anti-GroEL antibodies in cells and supernatants was used for assessment of cell lysis (two bottom panels). Mutants p9G2 and p4G8 are examples of clones that secrete Peb1 well (neutral mutants) and carry the insertions at positions H25 and G106, respectively. Mutants p4G10 and p4B7 have the insertion in the TAR1 region, whereas the insertion is in the TAR2 region in mutants p3D9 and p8C3. Mutant p1B6 has an insertion in the promoter region (at -87 nt) of the *fliC-peb1 *construct, which abolishes Peb1 expression. Wt Peb1 is encoded by plasmid pLA25 and pMCS3'UTR is the vector control. Cell-free culture supernatant (S) and whole bacterial cell samples (C) corresponding to 100 μl of bacterial culture were analyzed. Molecular weight markers in kDa are indicated on the left.

To identify regions in Peb1 putatively important for its transport, we constructed an exhaustive library of 5 amino acid insertion mutants. The target for the mutagenesis was the DNA fragment *fliC*5'UTR-*peb1*, in which *peb1 *is expressed from the P*_fliC _*promoter without the sequences encoding the N-terminal leader peptide and the putative processing site for signal peptidase II of Peb1. The coverage of the library was determined by DNA sequencing of the *fliC*5'UTR-*peb1 *fragment in all the 2037 library clones. The sequence of *peb1 *was successfully determined in 1827 clones and 1518 of these carried the pentapeptide inserts in the open reading frame of *peb1*. We found unique inserts at 178 sites in total along the entire amino acid sequence of Peb1, which is 233 amino acid residues in length. The longest region lacking mutations was only four amino acid residues in length (Figure 2A).

To identify secretion-deficient insertion mutants, we screened the 2037 library clones for Peb1 secretion by dot blotting of cell-free culture supernatants with anti-Peb1 antibodies. In the majority of Peb1-expressing mutants analyzed, the pentapeptide insertion in the amino acid sequence of Peb1 had no significant effect on secretion of Peb1 into the growth medium. Interestingly, we found 14 clones that showed impaired extracellular export of Peb1. When we analyzed the deduced amino acid sequence of these mutants, we noted that the pentapeptide insertions occurred at two confined regions in the Peb1 sequence: D42-V43 and S173-L180. The two regions were named Transport Affecting Regions 1 and 2 (TAR1 and TAR2; see Figure 2A). Six of the transport-affecting insertions were localized to the TAR1 region and eight to the TAR2 region.

We verified by SDS-PAGE and Western blot analysis using anti-Peb1 antibodies that the absence of the TAR mutants in the extracellular milieu was not due to impaired Peb1 expression or degradation of the protein. Results from the analyses of cells and cell-free culture supernatants of mutants representative of the entire data set are shown in Figure 2B. According to our analysis, the TAR1 mutant proteins were not degraded but expressed intracellularly at lower quantities than wt Peb1 (see top panel of Figure 2B), which compromised the judgement of the effect of these mutations on Peb1 secretion. The intracellular expression of TAR2 mutant proteins was equal to that of wt Peb1. Cell lysis was analyzed using antibodies against intracellular marker proteins as described previously [21] and was judged to be minimal (bottom panels of Figure 2B). The finding that the well-expressed TAR2 mutants were not found in the growth medium was additionally considered as an internal control for lack of leakage from or lysis of the cells.

When we compared the amino acid sequences of the inserted pentapeptides in TAR1, TAR2 and neutral mutants, we found that the neutral mutants carried insertions with similar residues as the secretion-affected mutants, but in locations outside the identified TAR regions. We deduced that the secretion-deficient phenotype of TAR1 and TAR2 mutants was not dependent on the type of sequence inserted and that the lack of extracellular localization by the TAR mutants was apparently due to the site of insertion (See Table 2 for examples).

**Table 2:** Sequence and localization of insertions present in Peb1 mutants

Localization^a)^ TAR1	Plasmid	Sequence	Localization^a)^ TAR2	Plasmid	Sequence	Localization^a)^ outside TAR1 and TAR2	Plasmid	Sequence
D42	p4G5	DAAAV	S173	p8C3	VRPHS	A2	pC1B11	DAAAA
D42	p4G10	CGRID	V174	p9B8	VRPHV	H25	p4G8	CGRTH
D42	p8E2	CGRID	D175	p2C3	CGRID	R89	pC18B11	CGRKR
D42	p7F4	VAAAD	L179	p3D9	LRPQL	I105	p9G2	GAAAI
V43	p4B7	CGRNV	L179	p3B7	LRPQL	T133	pC4E1	MPRQT
V43	p9D5	AAAAV	L180	p10B2	VRPQL	E220	pC6B3	LRPQE
			L180	p8F7	VRPQL	H221	pC7B5	CGRKH
			L180	p4B10	VRPQL	H221	pC18H10	AAAEH

a) amino acid residue after which the pentapeptide is inserted

Taken together, the exhaustive search for transport-affecting domains in Peb1 revealed two insertion-sensitive regions in the open reading frame of Peb1, TAR1 and TAR2, which affect its localization in the growth medium of *E. coli *MKS12.

### Effect of targeted mutations in TAR regions on Peb1 secretion

**Figure 3:**
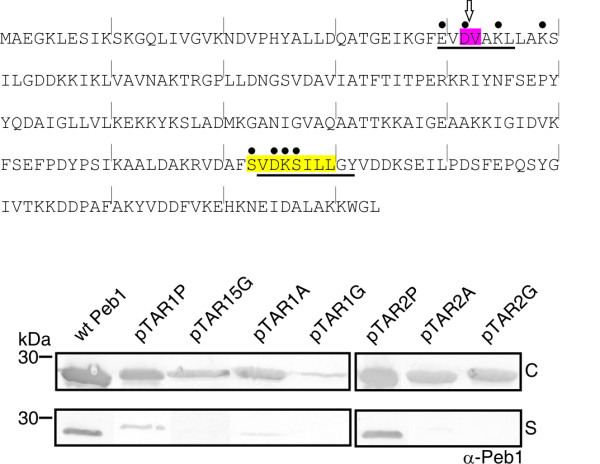
**Expression and secretion of site-specifically mutated Peb1**. The amino acid sequence of mature Peb1 shows the TAR1 and TAR2 regions in pink and yellow, respectively. Dots indicate sites for Ala point substitutions (encoded by plasmids pTAR1P and pTAR2P), the underlined sequences show sites for longer Ala and Gly substitutions (pTAR1A, pTAR1G, pTAR2A and pTAR2G), and the arrow indicates the site of a pentaglycine insertion (pTAR15G). Every tenth residue is indicated by a vertical line. The bottom panel shows whole cells (C) and cell-free culture supernatants (S) from strain MKS12 carrying the plasmids indicated above the panels analyzed by Western blotting using anti-Peb1 antibodies (see Fig. 2A and Table 1 for details regarding the plasmids). Wt Peb1 is encoded by the plasmid pLA25. Samples correspond to 100 μl of culture. Molecular weight markers in kDa are indicated on the left.

The results obtained from the mutant library of Peb1 indicated the two TAR areas in the protein as critical for its extracellular localization. To analyze these regions of Peb1 in more detail, we substituted all the charged and polar residues in the vicinity of and within the TAR1 region (Glu40, Asp42, Lys45 and Lys49) (encoded by plasmid pTAR1P) and in the TAR2 region (Ser173, Asp175, Lys176 and Ser177 (encoded by plasmid pTAR2P) with alanine (Figure 3). In addition, we generated an insertion of five Gly between Asp42 and Val43 in the TAR1 region (encoded by pTAR15G) (Figure 3). The Ala point mutations in TAR2 did not significantly influence the expression and secretion of the mutated Peb1 proteins, whereas in TAR1 the cellular concentration and, as a consequence, secretion levels of the Ala point mutants were slightly decreased. The cellular concentration of pentaglycine insertion mutant was also diminished and its transport was abolished (Figure 3).

We next assessed the impact of the TAR1 and TAR2 regions on Peb1 secretion by creating long Ala substitutions (Glu40 to Leu46 and Val174 to Tyr182) and for comparison, identical Gly substitutions were constructed (Figure 3). The mutant Peb1 proteins with nine residues long Ala or Gly substitutions in region TAR2 (encoded by pTAR2A and pTAR2G) were synthesized at the level of wt Peb1 but were not secreted, whereas the Peb1 mutants carrying seven-residue long Ala or Gly substitutions in TAR1 (pTAR1A and pTAR1G) showed a lower amount of Peb1 in cells compared to the wild type strain. The mutant with a long Gly substitution was not secreted and the mutant carrying a long Ala substitution was present in barely detectable amounts in the growth medium (Figure 3).

The results thus showed that selected Ala point substitutions in TAR1 and TAR2 regions do not affect Peb1 secretion significantly, whereas longer Ala substitutions in TAR1 and especially in TAR2 markedly impair Peb1 secretion. Also long Gly substitutions in the TAR regions and the insertion of five Gly in TAR1 affected transport of Peb1. In addition, the results showed that all manipulations performed in the TAR1 region, i.e. insertions, selected point substitutions as well as long substitutions, affected the amount of intracellular Peb1 whereas manipulations of the TAR2 region did not.

### Cytosolic Peb1 proteins are soluble and not subjected to aggregation

**Figure 4:**
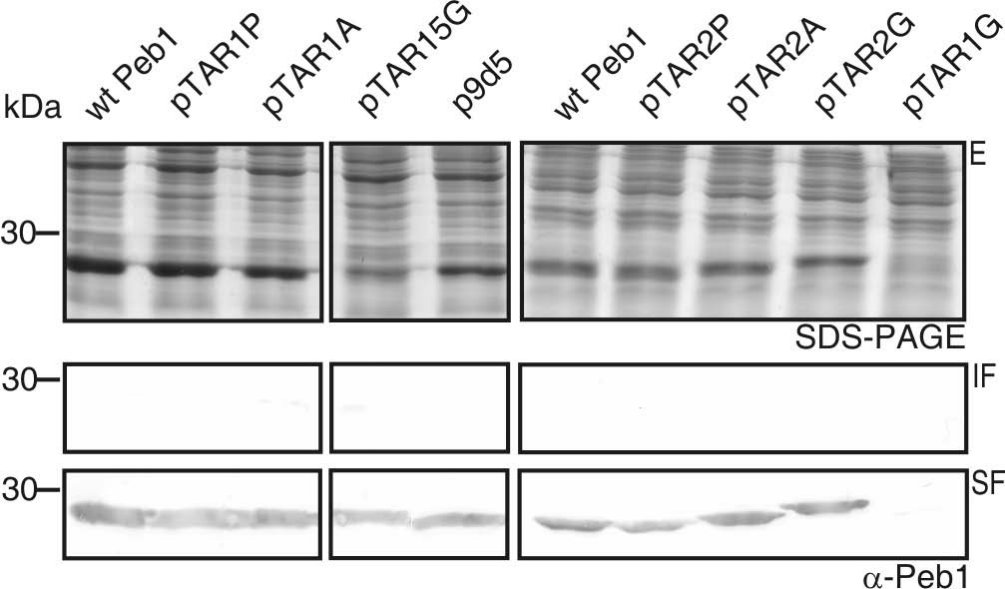
**Intracellular solubility analysis of wt and mutated Peb1 proteins**. The top panels show SDS-PAGE analysis of Peb1 expression in whole bacterial cells of MKS12 carrying the plasmids indicated above the panels. Cells were lysed and the cytoplasmic content was fractionated by ultracentrifugation as explained in methods section. Samples correspond to 100 μl of culture (top panel). The lower panels show Western blot analysis with anti-Peb1 antibodies of samples from the insoluble and soluble fractions. Samples correspond to 30 μl of culture. Plasmid pLA25 encoded wt Peb1, see Fig. 3 and Table 1 for details regarding the other plasmids. Molecular weight markers in kDa are indicated on the left. The letter E indicates expression, IF insoluble fraction, and SF soluble fraction.

To assess whether intracellular aggregation of mutant proteins was the reason for the lack of their presence in the growth medium, the cytoplasmic content of *E. coli *MKS12 expressing Peb1 and its mutants was fractionated by ultracentrifugation, separated by SDS-PAGE, and analyzed by Western blotting using anti-Peb1 antibodies (Figure 4). The experiment included wt Peb1, Peb1 with Ala- and Gly-substituted TAR regions, Peb1 carrying a pentaglycine insertion in TAR1, and one of the Peb1 mutants from the transposon library with the pentapeptide insertion in the TAR1 region (encoded by plasmid p9D5; see Table 2). The results showed that all the proteins that were expressed well in the cells were also found in the soluble fraction. The Peb1 mutant with a long Gly substitution in TAR1 (encoded by pTAR1G) was expressed very poorly and was therefore detected neither in the soluble nor the insoluble fraction, a fact hampering the analysis of its solubility. Conclusively, the results indicated that the lack of extracellular localization by Peb1 mutants in the analyses was not due to aggregation or proteolysis of the proteins in the cytoplasm.

### Subcellular localization of Peb1 mutants

**Figure 5:**
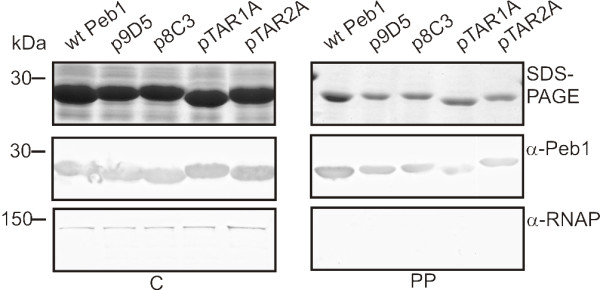
**Subcellular localization of Peb1 mutants**. SDS-PAGE (top panel) and Western blot (middle panel) analysis with anti-Peb1 antibodies of whole-cell samples and periplasmic extracts of MKS12 carrying the plasmids indicated. Cell lysis and cytoplasmic leakage was controlled by immunoblotting with anti-RNAP antibodies (bottom panel). Plasmids p9D5 and p8C3 carry insertions with no or minor effect on Peb1 expression and secretion efficiency, pTAR1A and pTAR2A carry long Ala substitutions in the corresponding TAR regions. Note the presence of Peb1 and its mutant derivatives in the periplasm of MKS12. The letter C indicates whole-cell samples, S cell-free culture supernatants, and PP periplasmic extracts. All the samples correspond to 100 μl of culture and molecular weight markers in kDa are indicated on the left.

To analyze the localization of Peb1 mutants, we prepared periplasmic fractions and cell samples of selected mutants which expressed Peb1 well but showed decreased Peb1 secretion. SDS-PAGE and Western blot analysis with anti-Peb1 antibodies showed that mutant Peb1 proteins with pentapeptide insertions or long Ala substitutions in the TAR1 and TAR2 regions were present in the periplasm at quantities almost equal to those of wt Peb1 (Figure 5). Western blot analysis with antibodies against the intracellular protein RNAP indicated that the presence of Peb1 and its mutants in the periplasmic samples was not due to cytoplasmic contamination (bottom panel in Figure 5). The results thus revealed that Peb1 mutants, which are well expressed in the cytoplasm but not secreted, are also transported almost as efficiently as wt Peb1 into the periplasmic space.

### Bioinformatics analysis of Peb1 and TAR regions

We performed sequence analysis of Peb1 in order to identify sequences important for its transport. The software programs SignalP, TatP, LipoP and Type-III effector prediction were used for prediction of putative signal sequences in the amino acid sequence of mature Peb1 (shown in Figure 3) and thus lacking the SecA-YEG -dependent signal sequence and the putative signal peptidase II recognition site [26-29]. We found no indication of the presence of a signal sequence related to SecA-YEG, Tat, lipoprotein or type 3 secretion system pathways in the mature Peb1. Interestingly, the SecretomeP [30] software predicted Peb1 to be a non-classically secreted protein.

**Figure 6:**
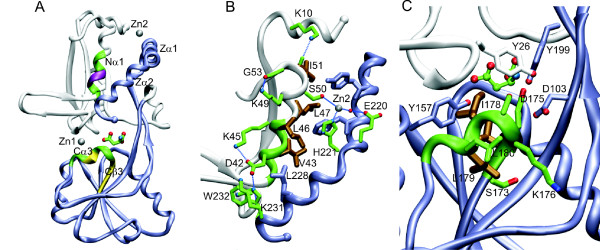
**Structure of Peb1**. (A) An overall view of the molecular structure. The first 100 residues are colored white, and the last 133 in greyblue, highlighting the two-domain structure of Peb1. The two pseudosymmetric domains fold into sandwiches of alternating alpha helices and beta strands. The N and C-termini are marked with spheres. The ligand binding site is situated at the domain interphase in the middle of the molecule, and a bound aspartate is shown as a stick model. Peb1 binds two zinc cations, which are indicated as grey spheres and marked with Zn1 and Zn2. Pentapeptide insertion sites in the TAR1 region are colored purple, and are situated in the first helix at the core of the N-terminal domain, Nα1. Pentapeptide insertion sites in the TAR2 region are colored yellow, and cover the end of the third beta-strand at the core of the second domain, and part of the following helix, Cβ3 and Cα3. The location of the long alanine substitutions in TAR1 and TAR2 are colored green. The two terminal helices of the protein are also marked, Zα1 and Zα2. In the indicated aspartate, carbons are colored green, oxygens red and nitrogens blue. The same color scheme is used in all atomic structure. (B) A detailed view of helix Nα1. The side chains of large hydrophobic residues of Nα1 are colored brown, and interactions with the surroundings are highlighted. (C) A close-up of area of the second cluster of disruptive substitutions at the center of the molecule, next to the the ligand-binding site. Residues of the long Ala substitution in this region are shown in green, except for three branched hydrophobic residues, V174, I178 and L179, that are drawn with thick brown lines. The neighboring residues are shown in atomic colors. Val174, Asp175, Ile178 pack tightly to each other to form the ligand-binding pocket along with Lys20, Ala65, Arg68, Ala82, Thr83, Phe84, Thr85, Arg90, Ala132, Thr133, Tyr157, Tyr199 from both domains.

Since the TAR regions were found to have a major impact on Peb1 secretion efficiency, we analyzed the sequence and the structure of Peb1 with emphasis on the TAR regions in order to identify possible structural aspects important for secretion. Mature Peb1 is a globular protein of 233 amino acids that folds into two domains both comprising of a five-stranded beta sheet surrounded by alpha helices, and it binds two Zn^2+ ^cations (Figure 6A). The TAR1 and TAR2 regions coincided with the first helix of the N-terminal core structure (Nα1) (Figure 6A, B) and with the end of the third C-terminal beta strand and the following helix (Cβ3-Cα3) (Figure 6A, C), respectively. The structural analysis here considered only the Ala substitutions since the pentapeptide insertions generated by the transposon mutagenesis are challenging to analyze in structural terms and interpretation of the effects of the Gly substitution in TAR1 was not reliable due to the decreased Peb1 expression (See Figure 3).

The helix Nα1, that covers the TAR1 region, is a 14-residue long, straight and strongly amphiphilic helix that interacts via Ser50 with one zinc cation but is not part of the ligand-binding site of Peb1. The polar residues of Nα1 form strong interactions with the two terminal helices of Peb1, Zα1 and Zα2, and the large apolar residues pack tightly to Zα2. The helix structure is stabilized by several hydrogen bonds (see Figure 6B). By the number of polar and apolar helix-helix interactions, the interface between Nα1 and Zα1 and Zα2 is the strongest in the whole protein. Substitution of all polar residues in and close to TAR1 by alanines (pTAR1P) did not significantly interfere with Peb1 transport, whereas a seven-residue run of alanines covering TAR1 (pTAR1A) did so, analogously to the results from the six amino acid transposon insertions (as shown in Figure 2 and 3).

The TAR2 region of Peb1 is located in the very center of the protein covering the end of Cβ3 and the beginning of Cα3 (see Figure 6A and 6C). It forms the ligand-binding pocket (residues Val174, Asp175, Ile178) along with numerous other residues from both domains. The results from Ala substitutions in TAR2 were similar to those in TAR1: the substitution by nine consecutive alanines compromised secretion, while changes of only the polar residues did not (see Figure 3). Sequence comparison between the secretion-deficient Peb1 constructs and those exhibiting a wt secretion level pointed towards an importance of the bulky hydrophobic residues Val174, Ile178, Leu179 and Leu180 for Peb1 secretion. Except for Leu180, these are fully buried residues, with solvent exposed surface areas smaller than 3% of their total area. Taken together, the structural analysis of TAR regions showed that the TAR1 region comprises a densely packed and highly stable part of Peb1 and the TAR2 region is located in the center of protein forming the ligand-binding pocket along with several other residues. We suggest that the unifying feature of the TARs is their crucial role for proper folding of Peb1, which apparently is important for the export across the OM.

## Discussion

When the Peb1 protein of *C. jejuni *is expressed without its native N-terminal signal sequence and without the putative signal peptidase II cleavage site in *E. coli *MKS12 (Δ*fliCfliD*), it is still efficiently secreted into the growth medium. The extracellular presence of Peb1 in MKS12 is not due to leakage from or lysis of the cells, as we have demonstrated previously [21]. Here, our aims were to analyze the molecular mechanisms behind the secretion competency of the heterologous model protein Peb1 in the *E. coli *host in order to broaden our understanding of factors that may affect secretion of proteins in *E. coli*. A detailed knowledge of mechanisms of extracellular transport of heterologous proteins in *E. coli *is of great importance in the development of secretion systems for biotechnological applications [4].

The present work shows that secretion of mature Peb1 into the growth medium of *E. coli *is facilitated also in the absence of the flagellar machinery as shown in the MKS12 derivative MKS111ΔHBB, a strain which completely lacks the flagellar secretion complex. The hypothesis of a flagellar-independent secretion mechanism was further supported by the fact that fusion of an N-terminal FliC fragment to the N-terminus of Peb1 did not interfere with its secretion. Our finding that Peb1 is also present in the periplasm strongly indicates that the protein is secreted by a mechanism that involves at least two export steps, one across the CM to the periplasm and another across the OM. This excludes secretion dependent only on the flagellar machinery, which is known to proceed from the cytoplasm to the extracellular milieu without involvement of a periplasmic step [22].

A comprehensive pentapeptide scanning mutagenesis of Peb1 was performed to decipher transport affecting regions in the molecule itself, and the results were confirmed by site-specific mutations. Importantly, modifications of only two distinct areas, named TAR1 and TAR2, in Peb1 affected secretion. This phenomenon was not due to insolubility or aggregation of the mutant proteins in the bacterial cytoplasm as shown by cell fractionation analysis. However, manipulations in the TAR1 region affected the intracellular amount of Peb1, whereas no such effect was observed after mutagenesis of the TAR2 region. We anticipate that the reduced amount of Peb1 seen in the cells of TAR1 mutants is due to decreased expression levels. As no degradation products of Peb1 were detected, we assume that significant proteolysis of Peb1 mutants did not occur. The results showed that also well-expressed, secretion-deficient Peb1 mutants were present in the periplasmic space. This finding strongly indicated that the export of the TAR1 and TAR2 mutants across the CM was unencumbered, whereas their further export across the OM was compromised and led to the observed lack of Peb1 mutants in the culture supernatant.

The secretion-deficiency of TAR mutants was studied also by analyzing the Peb1 structure. The effect of TAR1 mutations could lie in the importance of the hydrophobic surface packing toward the rest of the protein. Native-like amphiphilicity of Nα1 may be needed for the folding of Peb1, and inability to fold may be the basic cause for secretion defects. The importance of TAR2 in secretion is apparently related to the stability of the protein, as TAR2 is located in the center of Peb1 and forms the ligand-binding pocket along with several other residues. We suggest that the unifying feature of the TARs is their crucial role in proper folding. We speculate that Peb1 remains unfolded or is only partially folded during its export across the CM to the periplasm since mutagenesis of TAR regions did not affect this step. Correct or at least partially correct folding of Peb1 may, on the contrary, be important for its export across the OM into the growth medium.

Searches in the literature and publically available databases revealed that the host strain MKS12 used in this work, a Δ*fliCfliD *-derivative of *E. coli *MG1655, expresses at the culture conditions used only three characterized protein transport systems [31-34]. These are the SecA-YEG and Tat systems, which export proteins with cognate cleavable N-terminal signal sequences across the CM [35], and the flagellum-specific secretion system responsible for assembly of the flagellar filament [36]. As indicated by the bioinformatics analysis of the Peb1 amino acid sequence, none of these are apparently involved in *E. coli *-based Peb1 secretion. The SecA-YEG -independent secretion was here experimentally studied by analysis of periplasmic and cell samples of Peb1-expressing *E. coli *grown with or without subinhibitory concentrations of azide, which is known to inhibit protein export by the pathway [24].

Other well-characterized pathways dedicated for protein secretion in Gram-negative bacteria are normally not expressed, or the genes are fragmented, deleted or totally lacking in MKS12 as described below. The type 1 secretion system of *E. coli*, which is responsible for secretion of the toxin hemolysin in pathogenic *E. coli*, is not found in MKS12. The strain carries the genes encoding a hemolysin-like protein named HlyE/SheA/ClyA secreted into the medium by a so far unidentified mechanism in H-NS mutants [37], which MKS12 is not. The genes encoding a putative type 2 secretion system are present in MKS12, but the transcription of the two operons encoding the majority of the type 2 secretion system components is repressed by the H-NS protein expressed in MKS12 [38]. The genes encoding a pathogenesis-related type 3 secretion system are not present in MKS12 [32,39]. The alternative cryptic type 3 secretion system called ETT2 present in some pathogenic and commensal *E. coli *strains as well as the ancestral gene cluster encoding the alternative flagellar system Flag-2 in 20% of *E. coli *strains are both nonfunctional in MKS12 [39,40]. The strain possesses at least 16 chromosomal genes related to the type 4 secretion system, but the genes are not expressed in the culture conditions used in the study [41]. The type 5 secretion system is represented in *E. coli *K-12 strains only by the SecA-YEG -dependent autotransporter protein called antigen 43, which facilitates its own transport to the surface of the cell [42]. Finally, no orthologs of the type 6 secretion system proteins are present [43], the type 1 fimbria genes have been site-specifically deleted in MKS12 [44], and the curli-encoding operons are not expressed in MKS12 at the growth conditions used [45].

ABC transporters are CM-spanning proteins that energize the transport of their substrates by binding and hydrolysis of ATP. In bacteria, the ABC exporters facilitate the export of various compounds from the cytoplasm and the more common ABC importers import essential nutrients that are delivered by specific PBPs [15]. In *E. coli*, almost 80 ABC importer and exporter systems have been reported. These frequently transport only small molecules [13] and are probably not responsible for the efficient secretion of Peb1, which is a full-length globular protein of 233 amino acids.

The transport mechanism of the aspartate/glutamate -binding PBP Peb1 appears to differ markedly in *E. coli *from that of its structurally known homologs HBP and GlnBP from *E. coli *[46,47] and Debp from *Shigella flexneri *[48]. These ABC-transporter associated PBPs are all exported by the SecA-YEG pathway involving a cleavable N-terminal signal sequence. The sequence identity between Peb1, HBP, GlnBP and Debp is low (27% and 22%) and at the structural level, root mean square deviation values calculated between mature Peb1, HBP and Debp are only 2.2 Å since the orientation of the domains relative to each other varies between structures. Hence, the overall structural similarity of Peb1 with *E. coli *PBPs is probably not the reason for its efficient secretion in the heterologous *E. coli *host.

Our results show that in *E. coli*, mature Peb1 is present in the extracellular milieu but cannot be visualized on the surface of strain MKS12 (pLA25) with indirect immunofluorecence microscopy using anti-Peb1 antibodies or by electron microscopy (unpublished data) indicating that blebbing of OM vesicles [49] may not be involved in the secretion. Our results also show that Peb1 can be exported to the periplasm independently of the flagellar machinery, without a SecA-YEG-dependent N-terminal signal sequence independently of the Sec pathway, and without a signal peptidase II recognition site. Taken together, we conclude that Peb1 may be secreted by a still unknown secretion route, a conclusion supported by our result from software analysis of the Peb1 sequence indicating that the protein is secreted by a non-classical pathway.

Our findings are indirectly supported by a line of independent reports. According to Hu and colleagues [31] one third of the 4225 protein-encoding genes of *E. coli *K-12 are functionally unannotated, a fact which makes it possible to reveal novel mechanisms even in this well-studied organism. Proteins denoted as non-classically secreted are found extracellularly in various bacteria despite the absence of a defined secretion signal or motif [50,51]. Frequently, these proteins called moonlighting proteins have novel extracellular functions in addition to their well-known roles in the cytoplasm [52]. Comparison of the extracellular proteomes of *E. coli *K-12 and BL21 revealed a large number of proteins, both cytoplasmic and periplasmic, to be released by unknown mechanisms into the growth medium when cells were grown at high cell density [53]. We assume that Peb1 is secreted in *E. coli *by a still uncharacterized two-step pathway and that the structurally significant TAR- regions affect the secretion. We are currently exploring this possibility further.

## Conclusions

We have previously reported that the diagnostically interesting model protein Peb1 is efficiently secreted, without the sequences for SecA-YEG dependent secretion and signal peptidase II, into the growth medium of the secretion-competent strain *E. coli *MKS12 (Δ*fliCfliD*). Here, we analyzed the secretion mechanism further and noticed that Peb1 was also transported independently of the flagellar secretion machinery. We found, by construction and analysis of an exhaustive library of Peb1 insertion mutants, that mutagenesis of two segments called TAR1 (residues 42 and 43) and TAR2 (residues 173 to 180) prevented Peb1 secretion individually. Site specific mutagenesis of the TAR regions confirmed the importance of these regions for Peb1 secretion, and we verified that the secretion deficiency of Peb1 mutants was not due to insolubility or aggregation of the proteins in the cytoplasm. The mutant proteins were present in the periplasm as well as in the cells of MKS12 and we deduced that mutagenesis of TAR regions did not affect export of Peb1 across the CM, whereas its export over the OM was markedly impaired. We conclude that the localization of the mature model protein Peb1 in the growth medium of *E. coli *is due to an active secretion process, which involves two export steps, one across the CM into the periplasmic space and another across the OM. The export across the OM apparently requires proper folding of the TAR regions since mutagenesis of the regions affected Peb1 export across the OM and thereby presence of the mutant proteins in the extracellular milieu. Bioinformatics analysis of the Peb1 sequence indicated that the protein is transported by an unknown, non-classical pathway, which is a topic for further studies.

## Methods

### Bacterial strains and growth conditions

Bacterial strains and plasmids used in this work are described in Table 1. *E. coli *strains were cultivated by shaking in Luria broth [54] or on Luria agar plates for 16 h at 37°C supplemented with antibiotics (tetracycline 12.5 μg/ml, streptomycin 100 μg/ml, ampicillin 100 μg/ml) when appropriate. For TCA-precipitation and periplasmic isolation, the bacteria were further inoculated (1:30) into 3 ml and 12 ml, respectively, of N-minimal medium [54] supplemented with 0,2% casamino acids and appropriate antibiotics, and cultivated statically for 16-24 h at 37°C for optimization of protein secretion as we have described previously [21]. For intracellular solubility assays, the strains were cultivated by shaking in Luria broth supplemented with appropriate antibiotics for 18 h at 37°C followed by dilution 1:100, and cultivated without agitation for 20 hrs at 37°C in 20 ml Luria broth with antibiotics. For analysis of the effect of subinhibitory concentrations of NaN_3 _on Peb1 export, the strains were grown as described but in the presence of 3 mM NaN_3_.

### Antisera and reagents

Rabbit anti-Peb1 antiserum (diluted 1:50 000 for immunoblot analysis) and anti-flagellum antiserum (diluted 1:2000) were available from the previous work [21]. For control of cell lysis, polyclonal anti-GroEL antibody was used at a dilution of 1:10 000 (Sigma-Aldrich) and mouse monoclonal anti-RNAP at a dilution of 1:10 000 (Neoclone). Alkaline phosphatase-conjugated secondary antibodies were obtained from DakoCytomation and diluted to a concentration of 1.5 μg/ml (1:1000). All antibodies were diluted in 1% BSA/PBS. Reagents for recombinant DNA techniques and PCR were mostly obtained from Promega. For PCR cloning and mutagenesis Pfu DNA polymerase (Promega) was used. Colony PCR was performed using Dynazyme II DNA polymerase (Finnzymes). Primers used in this study were purchased from Medprobe and are shown in Table 3.

**Table 3:** Primers used in this study

Primer	Application	Sequence 5'-3'
Peb1RXbaI	PCR	GGCTCTAGATTATAAACCCCATTTTTTCGC
5'UTRfwBglII	PCR	GCCAGATCTGCGGGAATAAGGGGCAGAG
K1012R	PCR	GGTCTAGATTAACCCTGCAGCAGAGACA
FliC183XbaIR	PCR	GGTCTAGATTAAGTGGTAACTGTATCGTTAT
K1008OF	PCR	GGCGATCGGCGGGAATAAGGGGCAGAG
ORpLA15	PCR	GCCGCATGCCGATAAACAGCCCTGCGTTA
pBRAhdIF	Sequencing	GCCTTCGTTCATCCATAGTTGCCT
pLA21R	Sequencing	GCCCAGTTAATCAGGTTACAACGATT
NotI mini	Sequencing	TGCGGCCGCA
028R	Sequencing	CAATTCAACTTGTAGGCCTGATA
peb1/pLA25FIN	Construction of: pLA32	GATCACTCAAAATAATATCAACAAGGCAGAAGGTAAACTTGAGTC
peb1/pLA25RIN	pLA32	GACTCAAGTTTACCTTCTGCCTTGTTGATATTATTTTGAGTGATC
HotNIF	pTAR1P	TAGCTGTTGCCGCATTGCTAGCTGCAAGTATATTGGGTG
HotNIR	pTAR1P	GCAATGCGGCAACAGCTACTGCGAAACCTTTAATTTCAC
HotCIF	pTAR2P	CGTTTGCTGTAGCCGCAGCAATATTGTTAGGTTATGTGG
HotCIR	pTAR2P	ATATTGCTGCGGCTACAGCAAACGCATCAACTCTTTTAG
PebGlyF	pTAR15G	GGCGGTGGTGGGGGCGTTGCCAAATTGCTAGCTAAAAG
PebGlyR	pTAR15G	GCCCCCACCACCGCCATCTACTTCGAAACCTTTAATTTC
PebAla1F	pTAR1A	GCAGCAGCGGCGGCCGCAGCGCTAGCTAAAAGTATATTGGGTG
PebAla1R	pTAR1A	CGCTGCGGCCGCCGCTGCTGCGAAACCTTTAATTTCACCTGTTG
PebGly1F	pTAR1G	GGCGGCGGTGGTGGCGGTGGCCTAGCTAAAAGTATATTGGGTG
PebGly1R	pTAR1G	GCCACCGCCACCACCGCCGCCGAAACCTTTAATTTCACCTGTTG
PebAla3F	pTAR2A	GCAGCCGCAGCAGCAGCGGCAGCGGCGGTGGATGATAAAAGTGAAATTTTG
PebAla3R	pTAR2A	CGCCGCTGCCGCTGCTGCTGCGGCTGCAGAAAACGCATCAACTCTTTTAG
PebGly3F	pTAR2G	GGCGGCGGTGGTGGCGGGGGCGGTGGTGTGGATGATAAAAGTGAAATTTTG
PebGly3R	pTAR2G	ACCACCGCCCCCGCCACCACCGCCGCCAGAAAACGCATCAACTCTTTTAG

### Construction of bacterial strains

Bacterial strains used in this work are described in Table 1. The *E. coli *strains MKS111, MKS111ΔHBB and MKS12P*_lac_peb1 *were constructed from *E. coli *strain MKS12 [21] by site-specific mutagenesis using the *pir*-dependent suicide vector pCVD442 as described earlier [55]. For the construction of MKS111, a DNA fragment covering 2348 bp (nucleotides1128017-1130365) was amplified by PCR to contain an in-frame deletion of *flgM *(367 bp, nucleotides1129426-1129059). Chromosomal deletions of *flgDEFGHIJ *and *fliFGH *were performed to create the *E. coli *strain MKS111ΔHBB using the same principle as above. The genes *flgDEFGHIJ *were first deleted from the chromosome of MKS111 using a PCR-amplified fragment covering 6782 bp (nucleotides 1130748-1137529) containing a deletion of *flgDEFGHIJ *(4905 bp, nucleotides 1131696-1136600). From this strain *fliFGH *was then chromosomally deleted by the same principles; the DNA fragment of 5589 bp (nucleotides 2010111-2015699) contained the deletion of *fliFGH *(3329 bp, nucleotides 2011212-2014541). For the construction of strain MKS12P*_lac_peb1*, a 3066 bp DNA fragment was amplified by PCR to contain *fliC *flanking regions (nucleotides 199936-2000133 and nucleotides 2001784-2003814 with the exception of the *fliD *deletion, nucleotides 2002082-2003109), P*_lac_*, *fliC *5' UTR and *peb1 *lacking its signal sequence. This fragment was used for substitution, by homologous recombination, of *fliC *in strain MKS1 [56]. Primers used in mutagenesis were designed on the basis of the nucleotide sequence of *E. coli *MG1655 and, for the P*_lac _*promoter, *E. coli *DH1 (GenBank Accession numbers NC 000913 and CP001637.1) [57]. Chromosomal DNA of *E. coli *MG1655 Δ*fimA-H*, also called AAEC072A, and plasmid pTSF12 [58], were used as templates for PCR amplifications. The genotypes of the mutants were confirmed by PCR and Southern blot hybridization.

### Construction of plasmids

Plasmids used in this work are described in Table 1. The authenticity of the construct has been verified for each plasmid by sequencing. Plasmids pMCS3'UTR and pPeb15'3' were available from the previous work [21]. Our former results indicated that the 5' UTR of *fliC *is important for secretion of Peb1 in MKS12, whereas expression of *peb1 *from e.g. the promoter and 5'UTR of the ß-lactamase-encoding gene rendered Peb1 in the cytoplasm of MKS12 [21]. Therefore, in the current study all the *peb1 *constructs were generated to maintain the sequence corresponding to the 5' region of *fliC *mRNA and thereby facilitate secretion of the gene product. To construct pLA25 a DNA fragment containing the *peb1 *gene, without the sequence encoding the leader peptide, fused to the 173 bp 5'UTR of *fliC *that includes P*_fliC _*, was constructed by PCR amplification from pPeb15'3 with primers containing *Bgl*II and *Xba*I restrictions sites (primers 5'UTRfwBglIl and Peb1RXbaI). This DNA fragment was ligated into the *Bgl*II-*Xba*I digested pMCS3'UTR and the ligation mixture was introduced into *E. coli *MKS12 cells by electroporation. To construct the *fliC*5'UTR-*peb1 *target for the *in vitro *transposition reaction, the plasmid pLA26 was generated. The 3'UTR of *fliC *in pLA25 was removed by *Sca*I-*EcoR*I digestion and the cohesive ends were subjected to a blunting reaction with Mung Bean Nuclease at a concentration of 5 units per μg DNA for 10 min at 37°C. The blunt ends were ligated, and the ligation mixture was introduced by electroporation into *E. coli *DH10B cells. To construct plasmid pLA31, a fragment containing P*_fliC _*and the sequence encoding the N-terminal 183 residues of FliC was amplified from chromosomal DNA of *E. coli *AAEC072A (primers 5'UTRfwBglIl and FliC183XbaIR, respectively). The *Bgl*II-*Xba*I-digested PCR fragment was cloned into pMCS3'UTR and subsequent ligation mixture was introduced into MKS12 by electroporation. To create pLA32 a PCR-generated DNA fragment containing the *fliC *5'UTR and 60 nucleotides from the 5' end of the *fliC *gene (p20FliCGfp5'3' [21] as template, primers 5'UTRfw*Bgl*II and peb1/pLA25RIN) was fused to a PCR-generated *peb1 *fragment (pPeb15'3' as template, primers peb1/pLA25FIN and Peb1R*Xba*I). This fusion fragment was cloned into pMCS3'UTR using appropriate restriction sites (*BglI*I and *Xba*I) and the ligated plasmid pLA32 was introduced into *E. coli *MKS12. For pTAR constructs, see Table 1.

### Precipitation of protein in growth media

To analyze the presence of Peb1 and mutant proteins in the growth medium, we precipitated culture supernatants with TCA as described previously [21]: 1,5 ml of culture in N-minimal medium was centrifuged in a tabletop centrifuge (Biofuge 13, Heraeus Instruments) at 13 000 rpm for 15 min at 4°C. The supernatant was recentrifuged, and 1ml of the secondary supernatant was precipitated with 10% TCA on ice for 30 min. Precipitates were pelleted (as above) and washed twice with ice cold ethanol/ether (1:1). Precipitated protein was allowed to dry before resuspension into loading dye and boiled for 5 min. An amount corresponding to 1 ml original cultivation when analyzed by SDS-PAGE and to 100 μl cultivation when analyzed by western blotting, respectively, was loaded onto the gel. Cell samples were generated by pelleting the cultivation followed by a wash with PBS. Cells were then resuspended into appropriate volume of loading dye for the analysis of the amount corresponding to bacterial cells from 100 μl culture, and boiled.

### Isolation of periplasmic contents

To assess the presence of Peb1 and mutant proteins in the periplasmic space, contents of the periplasm were isolated. Bacteria were grown overnight in Luria broth and diluted in N-minimal medium (1:30) for further cultivation for 20 hrs without agitation at 37°C. Cells were then pelleted using a F21-rotor (FiberLite^®^, Piramoon Technologies, Inc.) in a Sorvall RC-5B centrifuge (Du Pont Instruments) at 10 000 g (15 min, 4°C). Cells were resuspended in 4 ml 20% glucose/g wet cells and incubated 5 minutes at RT. Next, the sample was centrifuged for 5 min at 13.000 rpm (RT) in tabletop centrifuge (PICO 17, Thermo Scientific). The pellet was further resuspended in 1 ml ice cold 10 mM Tris/20 Mm EDTA (pH 7.5)/g cells, incubated on ice 10 min and centrifuged additionally for 5 min (13 000 rpm, 4°C). The remaining supernatant represented the periplasmic content. All SDS-PAGE and Western blot analyses contained periplasmic contents corresponding to 100 μl original culture as to be comparable with cell and TCA-precipitation samples.

### Intracellular solubility assay

To separate the soluble and insoluble protein fractions of the bacterial cytoplasm, we performed fractionation by ultracentrifugation. The expression sample (100 μl) was removed after 18 h cultivation and centrifuged for 10 min at 10 000 × g. The pellet was resuspended in SDS-PAGE sample buffer and boiled for 5 min prior to loading onto gel. The remaining portion of the overnight culture was centrifuged for 10 min 3500 rpm in a tabletop centrifuge and washed once with 10 ml PBS. The pellet was resuspended in 5 ml PBS and sonicated (in a Vibracell apparatus, Sonics & Materials Inc., tip model CV26) 10 × 10 sec at 20% amplitude to lyse the cells. The cytoplasmic content was cleared by centrifugation in a tabletop centrifuge (Biofuge 13, Heraeus Instruments) for 15 min at 6000 rpm (4°C). The supernatants were subsequently fractionated by ultracentrifugation for 1 h at 4°C with a Ti 50 rotor for 100 000 × g (Beckman L-60 Ultracentrifuge). The supernatant consisting of the soluble fraction of the cytosolic content was removed and the pellet, representing the insoluble fraction, was resuspended in PBS. Samples for SDS-PAGE analysis, corresponding to 100 μl cultivation, were prepared by adding SDS-PAGE sample buffer and boiling for 5 min before loading onto gel. In Western blot analysis of soluble and insoluble fractions the samples corresponded to 30 μl cultivation.

### Western blotting and SDS-PAGE analysis

Western blotting and SDS-PAGE analyses were carried out essentially as described using 15% SDS-polyacrylamide gels [21]. In Western blotting, nitrocellulose membrane with pore size 0.2 μm or 0.45 μm (Protran Whatman^®^, Schleicher&Schuell), was used. Blocking with 2% BSA/PBS was done for 18 h at RT, and detection employed appropriate antibodies.

### Generation of the insertion mutant library

Insertion mutagenesis of the DNA fragment *fliC*5'UTR-*peb1 *was accomplished using the Mutation Generation System (Finnzymes) that employs MuA transposase to insert an artificial transposon randomly into a target plasmid [59]. Upon transposon elimination by restriction digestion and plasmid recirculation by ligation, the system yields fifteen base pair insertions in target plasmids [60,61]. The *in vitro *transposition reaction was essentially done according to instructions supplied by the manufacturer. The reaction contained 100 ng M1-CamR transposon DNA as a donor, 480 ng plasmid pLA26 as a target, 0.2 μg MuA transposase, 25 mM Tris-Hcl, pH 8.0, 100μg/ml BSA, 15% (w/v) glycerol, 100 mM NaCl, 0, 05% Triton X-100 and 10 mM MgCl_2 _in a total volume of 25 μl. The reaction was conducted at 30°C for 3 h. The DNA was purified, precipitated, and transformed into *E. coli *DH10B cells by electroporation. The plasmid DNA was isolated from a pool of 90 000 colonies using the Plasmid Maxi Kit (Qiagen). Subsequently, the *fliC*5'UTR-*peb1 *target fragment was removed by *Bgl*II-*Xba*I digestion and ligated with equally digested pMCS3'UTR. The body of the transposon was removed by *Not*I digestion after which the plasmids were recirculated by ligation. This pool of mutated plasmids represented the insertion mutant library.

### Screening of the mutant library

For screening purposes, plasmids of the insertion mutant library were introduced into *E. coli *MKS12 cells by electroporation and transformants were randomly picked for screening from selection plates. Primary screening of the mutant library was based on dot blotting of the cleared supernatant from cultures of library clones. In total 2037 transformants were cultured without agitation on 96-well microtiter plates in 200 μl LB supplemented with tetracycline at 12.5 μg/ml for 16 h at 37°C and 5 μl overnight culture was subsequently transferred into 200 μl N-minimal medium supplemented with 0.2% casamino acids and tetracycline (12.5 μg/ml) and cultured statically for 20 hours. The cells were pelleted by centrifugation of the microtiter plate for 15 minutes at approximately 2000 × g (Heraeus Megafuge 1.0, rotor model 2705, microtiter carrier model 2708) and the remaining supernatants were cleared by centrifuging 150 μl supernatant for an additional 15 min at 2000 × g. Next, 100 μl cleared supernatant was dot blotted onto nitrocellulose membrane (pore size 0.45 μm; Protran Whatman^®^, Schleicher&Schuell) using the Bio-Dot^® ^Microfiltration Apparatus (Bio-Rad Laboratories) according to manufacturers' instructions. The membrane was subsequently incubated in 2% BSA/PBS for 18 h at 20°C and thereafter the presence of Peb1 protein was detected with anti-Peb1 antibodies and alkaline phosphatase -conjugated secondary antibodies. In order to verify that Peb1 was expressed intracellularly, the cell pellets from the microtiter plates were washed with 100 μl PBS, repelleted (10 min for 1000 rpm, approx. 170 × g) and lysed by resuspension in SDS-PAGE sample buffer (30 μl) and boiling for 5 min. Samples equivalent to 30 μl of bacterial culture were analysed by SDS-PAGE. Secondary screening of clones expressing Peb1 intracellularly at the same level as wt Peb1 but showing decreased Peb1 export was performed by precipitation of cleared supernatants with 10% TCA and Western blotting with anti-Peb1 antibodies as described elsewhere [21]. Localization of the insertion site in the mutated *peb1 *genes was done by colony PCR using either forward (pBRAhdIF) or reverse (pLA21R) primer together with the *Not*I-miniprimer (Finnzymes, Table 3.) that recognizes the 15 bp insert originating from the transposition event. Ultimately the insertions' location was specified by sequencing (Institute of Biotechnology, University of Helsinki, Finland) using the appropriate primers (pBRAhdIF or 028R).

### Bioinformatic analysis

The amino acid sequence of mature Peb1 was analyzed for the presence of a secretion signal using the softwares SignalP [26], TatP [27], SecretomeP [30], LipoP [29] and Type-III effector prediction [28]. The protein structures were visualized with the program VMD [62]. The sequences of bacterial periplasmic amino acid binding proteins were aligned by ClustalX 2.0.5 [63] and the solvent exposed surface area per residue was calculated with the program GetArea [64].

### Accession numbers

The accession numbers [65] of the cited atomic coordinates are Peb1 [PDB:2v25], glutamine binding protein GlnBP from *E. coli *[PDB:1ggg], histidine binding protein HBP from *E. coli *[PDB: 1hsl], and glutamate/aspartate binding protein Debp from *S. flexnerii *[PDB: 2vha]. The cited amino acid sequence is Peb1 [GenBank: AAA02919.1]; the genome sequences are from *E. coli *MG1655 [GenBank: U00096], the parental strain of MKS12, and *E. coli *DH1 [GenBank: CP001637.1] [57].

## List of abbreviations used

(3'UTR): 3' untranslated region; (5'UTR): 5' untranslated region; (ABC): ATP-binding cassette; (CM): cytoplasmic membrane; (P*_fliC_*): *fliC *promotor; (OM): outer membrane; (PBP): periplasmic binding protein; (PBS): phosphate-buffered saline, pH 7,1; (BSA/PBS): bovine serum albumin in PBS; (RNAP): RNA polymerase β subunit; (SDS-PAGE): sodium dodecylsulphate polyacrylamide gel electrophoresis; (TCA): trichloro acetic acid; (wt): wild-type.

## Competing interests

The authors declare that they have no competing interests.

## Authors' contributions

LA carried out the generation and screening of the insertion mutant library, the precipitation of proteins, the isolation of periplasmic contents, the intracellular solubility assays as well as the SDS-PAGE and Western blot analyses. She also constructed the plasmids, participated in the study design and interpretation of data, and in drafting of the manuscript. KM carried out construction of the *E. coli *host strains and participated in revising the manuscript critically. LL carried out the structural analysis, participated in the study design and in revising the manuscript critically for important intellectual content. HS participated in the design and generation of the insertion mutant library and participated in revising the manuscript critically. BWW had the main responsibility for the study design, data interpretation and manuscript writing. All authors read and approved the final manuscript.
